# Supplementary material for: Increased basal ganglia volume in older adults with tinnitus
Source: Sci Rep. 2025 Nov 21;15:41303. doi: 10.1038/s41598-025-25065-6 (PMC12638945; doi:10.1038/s41598-025-25065-6)

**Increased basal ganglia volume in older adults with tinnitus**

Simón San Martin^2,6+^, Vicente Medel^3+^, Hayo Breinbauer^2^,

Carolina Delgado^2,4^, Paul H. Delano^1,2,5,6^*

1. Servicio Otorrinolaringología, Hospital Clínico de la Universidad de Chile, Santiago, Chile.
2. Departamento de Neurociencia, Facultad de Medicina, Universidad de Chile, Santiago, Chile.
3. Latin American Brain Health Institute (BrainLat), Universidad Adolfo Ibanez, Santiago, Chile.
4. Servicio de Neurología y Neurocirugía, Hospital Clínico de la Universidad de Chile, Santiago, Chile.
5. Biomedical Neuroscience Institute (BNI), Facultad de Medicina, Universidad de Chile, Santiago, Chile
6. Advanced Center for Electrical and Electronic Engineer (AC3E), Valparaiso, Chile.

*Correspondence:

Paul H. Delano

Av. Dr. Carlos Lorca Tobar 999, Independencia

Hospital Clínico Universidad de Chile

Santiago, 8380456

Chile

Email: [pdelano@hcuch.cl](mailto:pdelano@hcuch.cl)

+These authors contributed equally and share first author

**Supplementary material**


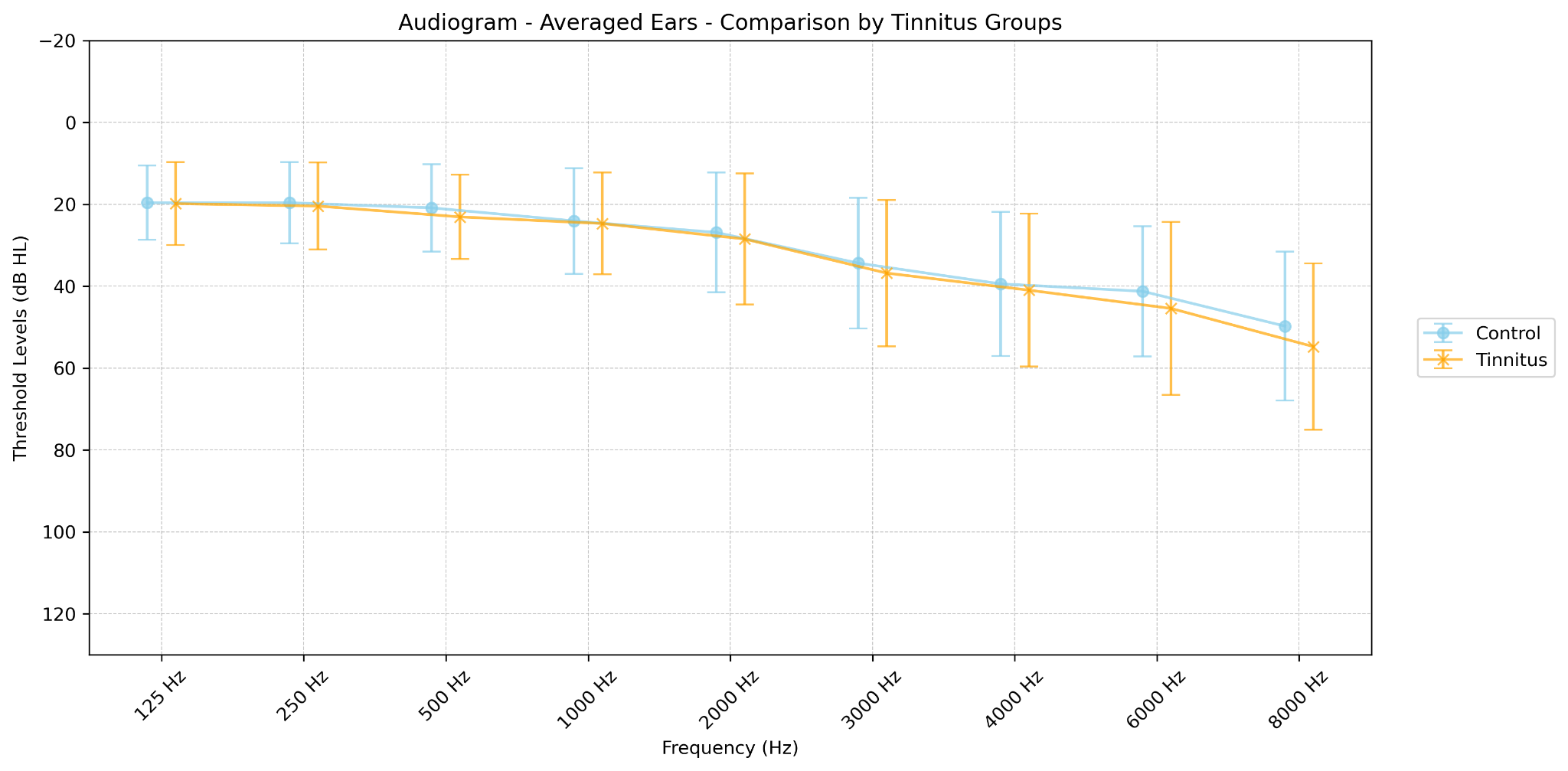


**Figure S1: Audiogram of averaged ears for each group compared.** Audiometric profiles for the tinnitus and control groups across tested frequencies (0.125 Hz to 8 kHz). Mean hearing thresholds (averaged across both ears) are plotted for each group, with error bars representing standard deviations. No significant differences were observed between groups at any frequency (p > 0.05, t-tests), as shown in the overlapping data distributions.

**Figure S2: Violin plot for LDL values in both groups.** Average loudness discomfort level (LDL) values (in dB HL) across tested frequencies (250 Hz to 4000 Hz) for the tinnitus and control groups (continuous lines). For subjects where no LDL was detectable due to audiometer output limits, putative values were imputed (110 dB HL at 250 Hz; 120 dB HL at 500-4000 Hz) to facilitate visualization of the distribution. Note that this imputation is for illustrative purposes only and was not used in the primary statistical analyses, which employed a counting approach for detectable LDL frequencies.
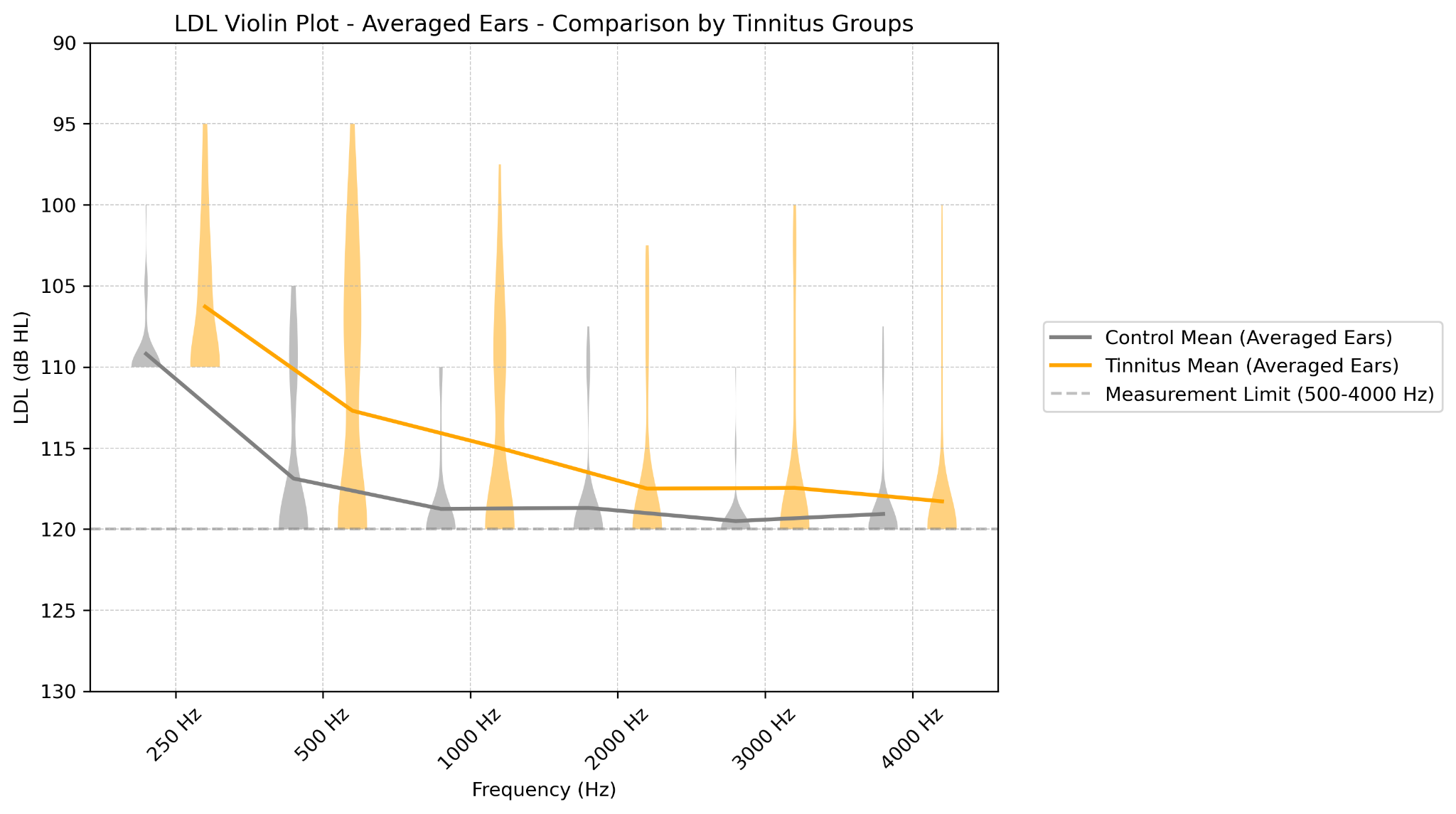

Supplement: Supplementary file 1 — Supplementary Material 1 [file 41598_2025_25065_MOESM1_ESM.docx]
